# Mood and Stress-Relief Supplement and OTC Drug Use among Polish Adults: A Comparative Study

**DOI:** 10.1192/j.eurpsy.2026.10429

**Published:** 2026-07-31

**Authors:** A. Brzozowska, J. Grabowski, M. Brosz

**Affiliations:** 1 Adult Psychiatry Scientific Circle, Division of Developmental, Psychotic and Geriatric Psychiatry, Department of Psychiatry; 2 Division of Developmental, Psychotic and Geriatric Psychiatry, Department of Psychiatry, Medical University of Gdańsk; 3 Institute of Sociology, Faculty of Social Sciences, University of Gdańsk, Gdańsk, Poland

## Abstract

**Introduction:**

The market for dietary supplements and over-the-counter (OTC) drugs intended for mood enhancement and stress reduction (“preparations”) is substantial. In the European Union, 10% of supplement users report taking them to alleviate depression, stress, or anxiety, with higher prevalence among women and adults aged 18–44 (Ipsos, 2022). Such popularity may reflect increased psychological distress, limited access to mental health care, mental health stigma, or limited trust in the healthcare system. Many of these preparations contain herbal ingredients with limited scientific evidence and potential safety concerns, including pharmacological interactions.

**Objectives:**

The study aimed to assess the prevalence and patterns of use of such preparations among Polish adults, and to compare users and non-users in terms of psychosocial factors, including coping strategies, help-seeking behaviours, and trust in physicians.

**Methods:**

A cross-sectional online survey was conducted in 2024 among 799 Polish adults. Respondents were classified as current, former or never users. Associations between preparation use and study variables were analysed using χ² or Fisher’s exact tests for categorical data, and Kruskal–Wallis or Mann–Whitney tests for ordinal data. Effect sizes were expressed as Cramer’s V or φ. Trust in physicians was assessed using selected items adapted from validated scales described in the literature.

**Results:**

Overall, 23.1% of respondents reported current and 37.2% past use of preparations. Supplements (79.9%) were more common than OTC drugs (22.3%). The most frequently used ingredients were *Withania somnifera* (ashwagandha), *Melissa officinalis*, *Valeriana officinalis*, *Humulus lupulus* (hops), *Hypericum perforatum* (St. John’s wort) and *Rhodiola rosea.* No significant differences were observed between groups in past or current use of formal mental health services (p=0.258). Users more often indicated psychotherapy (p=0.002), using preparations (p<0.001) and psychiatric consultations (p=0.001) as preferred future coping strategies. They reported higher global trust in physicians (p<0.001) and trust in the healthcare system (p=0.04), but lower ratings of doctors’ communication competency (p=0.002). Over half of those using both preparations and regular prescription medicines did not inform their doctor. Users declared less willingness (p<0.001) and less comfort (p=0.015) in discussing their doubts about the use of preparations with their doctor.

**Conclusions:**

Although users and non-users did not differ in formal mental health service utilization, they varied in future coping preferences, comfort in discussing product use, and certain aspects of trust in physicians. Addressing these communication barriers may support safer, evidence-based use of supplements and OTC medicines for mental health purposes.

**Disclosure of Interest:**

None Declared

